# Selective mortality during famine and plague events in medieval London

**DOI:** 10.1038/s41598-025-13198-7

**Published:** 2025-07-25

**Authors:** K. Godde, Sharon N. DeWitte, Julia Beaumont, Brittany S. Walter, Rebecca Redfern, Jelena J. Bekvalac

**Affiliations:** 1 School of HESBS, Moreno Valley College, Moreno Valley, CA 92551 USA; 2 Institute of Behavioral Science, University of Colorado Boulder, 80309 Colorado Boulder, USA; 3 Department of Anthropology, University of Colorado, Boulder, CO 80309 USA; 4 School of Archaeological and Forensic Sciences, University of Bradford, Bradford, UK; 5 Department of Defense, Defense POW/MIA Accounting Agency, Offutt AFB, 68113 Bellevue, USA; 6 London Museum, 150 London Wall, London, EC2Y 5HN UK; 7 School of History, Classics and Archaeology, Newcastle University, Newcastle upon Tyne, England, NE1 7RU UK

**Keywords:** Demography, Frailty, Imputation, Stable isotopes, Crisis mortality, Anthropology, Archaeology

## Abstract

Crisis mortality events such as disease epidemics and famines have long had major impacts on human health and demography, and clarifying variation in susceptibility to crisis mortality is crucial for enhancing our understanding of the larger-scale phenomena of human adaptation and biological variation. Previous bioarchaeological research using data from human skeletal remains has revealed variable patterns of biological, and by inference, social determinants of vulnerability to medieval famine and plague mortality. However, such previous work did not account for missing data and the potential associated biases. For this study, we apply a novel approach integrating dietary isotope, pathological, and demographic data into multinomial logistic regression analyses of mortality differentials during famine and plague events in medieval London, while controlling for missing data. The results suggest higher risks of famine and plague mortality for males and for individuals previously exposed to stressors, and higher risks of plague (but not famine) mortality for older adults. Further, we find evidence that protein-rich diets were protective against famine and plague mortality. Our findings highlight how various biological and social factors shaped risks of mortality in medieval London, even in the context of major mortality crises, which are often assumed to be indiscriminate in nature.

**Supplementary Information:**

The online version contains supplementary material available at 10.1038/s41598-025-13198-7.

## Introduction

Crisis mortality, a dramatic but temporary increase in mortality rate resulting from a single extraordinary factor, can be caused by a variety of events such as disease epidemics, famines, floods, and warfare^1–3^. Crisis mortality is believed to have dominated pre-industrial, agricultural population dynamics prior to relatively recent demographic transitions^4,5^ and it is still an impactful phenomenon in many living populations^6,7^. For example, we have witnessed myriad effects of the COVID-19 pandemic, such as reductions in sex-ratios at birth^8,9^ and decreases in life expectancy and variation thereof by racial/ethnic groups^10^. Thus, crisis mortality has had, and still has, the potential to powerfully shape human demography and biological variation over the short- or long-term.

Anthropological, historical, economic, paleogenomic, and epidemiological work on past mortality crises has examined various factors that shape vulnerability to mortality during these events (e.g., variation in nutritional status and physiological stress) and their longer-term consequences (e.g., changes in diet, genetics, health, survivorship, and migration). For example, exposure to the Great Chinese Famine (1958–1962) produced intergenerational vulnerability to tuberculosis^11^. The 1918 influenza pandemic disproportionately affected individuals previously exposed to physiological stressors^12^ and those with tuberculosis, leading to reductions in tuberculosis rates for several years after the pandemic ended^13^; individuals exposed to the 1918 flu *in utero* experienced negative outcomes with respect to educational attainment, physical disability, and income decades later^14^. The Black Death and later post-medieval plagues produced asymmetrical economic consequences that were negative in some affected areas and positive in others^15,16^. Medieval and post-medieval plague outbreaks also appear to have differentially affected men and women^17^ and disproportionately affected impoverished people in some areas^18^. More examples of variation in mortality during historic plague events are detailed below. However, there is still more to learn about these phenomena, particularly for more ancient events with fewer surviving sources of evidence. Such clarity is crucial for understanding human evolution; modern human biological variation; the social, economic, and political context of crises; and the range of outcomes that can result from crises.

### Medieval plague

There were many mortality crises, including famines and plague epidemics, during the medieval period in England (1066–1485 CE). Perhaps the best-known of these was the first wave of the Second Pandemic of Plague (the Black Death), which was caused by *Yersinia pestis* (the pathogen that continues to cause bubonic plague today) and swept across Afro-Eurasia in the mid-14th century CE, and affected England between 1348 and 1350 CE. Historical evidence suggests that the epidemic killed approximately 30–60% of affected populations in England^19^ The Black Death was soon followed by a second outbreak of plague in England in 1361 CE (the *pestis secunda*), killing an estimated 10–30% of affected populations^19^.

Bioarchaeological and historical work has provided some insights on variation in mortality patterns during the Black Death and subsequent outbreaks of plague. Bioarchaeological analysis of individuals who died during the Black Death in London has suggested that people with African heritage, and who therefore may have been marginalized, experienced elevated risks of death^20^. Individuals with skeletal biomarkers of stress apparently faced higher risks of mortality during the Black Death^21–24^ and a post-Black Death plague outbreak (perhaps the plague of 1361 CE)^19^. However, people with low levels of frailty (as indicated by a frailty index) in 17th -century CE Italy suffered higher risks of plague mortality^25^. Analyses of age patterns of mortality have revealed contradictory findings^23,26^which may be the result of different age-estimation methods, analytical approaches, or differences in sample size and composition. To date, there is no bioarchaeological evidence of significant sex differences in risk of plague death. However, historical evidence from England suggests higher mortality for men during the plague of 1361 CE^19^. Conversely, historical evidence from the Netherlands, c. 1349–1450 CE^17,27^ and epigraphic evidence from Kyrgyzstan, c. 1338–1339 CE^28^ suggest higher risks for women during medieval plague epidemics.

For this study, we focus specifically on the 1361 CE *pestis secunda *because the majority of existing bioarchaeological and historical research on medieval plague has focused on the Black Death, and we want to broaden the scope to include another relatively understudied but incredibly important outbreak. Based on historical data, see e.g^19^.,, the *pestis secunda* produced lower mortality rates than the Black Death (though mortality was still much higher than baseline), and a recent study by Sidhu et al.^29^ has produced evidence of genetically-driven attenuated virulence in *Yersinia pestis* within 100 years of the initiation of each of the three major plague pandemic (which began in the 6th, 14th, and 19th centuries, respectively). Therefore, it is important to examine, with as many lines of evidence as possible, mortality patterns across several different outbreaks during the larger Second Pandemic to better understand how plague, specifically, and infectious diseases more generally change after their initial emergence and how dynamics within human populations may be associated with evolutionary changes in pandemic pathogens.

### Medieval famine

Famine also substantially affected living conditions and mortality in medieval England. Although local food shortages appear to have been relatively common throughout the medieval period in England, there was variation in the severity and extent of famines, with a few that were incredibly severe and for which there are sufficient historical sources to infer overall mortality levels. Specifically, a series of back-to-back harvest shortfalls in 1256–1258 CE led to a prolonged famine through 1260 that produced mortality levels that might have been as high as 10–15%^30,31^, on par with the later Great Famine *c.* 1315–1317 CE^32,33^. Soon after the Great Famine, England and Wales were struck by the Great Bovine Pestilence, which killed over 60% of bovine populations and led to dairy scarcities that lasted into the 1330 s CE^34,35^. The period from 1321 to 1347 CE was characterized by relative stability with respect to crop yields and improved rural wages, but subsequently, two cool climatic periods produced losses in crop yields coinciding with the Black Death (1348–1350 CE in England) and the *pestis secunda* of 1361 CE^36^.

Previous analyses of skeletal stress markers among people who ultimately died during the Black Death in London in 1349 hint at the possibility that frail people were disproportionately killed during the earlier Great Famine, but individuals born after the Great Famine did not experience as severe a selective sweep and therefore may have had higher frailty on average than earlier cohorts, suggesting a link between famine experience and vulnerability (or not) to plague^37^. It is possible that individuals in their late 40 s and older at the time of the 1361 *pestis secunda* had survived through several previous highly catastrophic crisis events, while those who were ~ 15–40 years of age at the time of the plague outbreak might have benefitted from the relatively stable period prior to the Black Death. The experiences of these individuals, particularly during formative years, may have influenced their ability to survive the plague of 1361. Bioarchaeological studies suggest that early life stressors were associated with elevated risk of medieval famine death^37–39^and that females faced higher risk of famine mortality^40^.

Because we cannot confidently assign anyone included in our study to any particular famine, for this study we chose to pool all famine burials into a single “famine” category. However, we realize our analyses may produce results that are not purely reflective of outcomes for each individual medieval famine event. Dividing the famine sample into temporal subgroups (e.g., separating famine burials in the period 1250–1350 CE from the remainder that were presumably less severe) would make it difficult to discern whether any observed differences in famine mortality patterns over time is reflective of true variation in famine mortality by severity rather than an artifact of sample size.

### Stable isotope analysis

Prolonged starvation may manifest biochemically in tissue and be detectable via analysis of dietary stable isotopes (e.g., carbon and nitrogen). Without sufficient protein consumption to maintain tissues, such as during nutritional stress (i.e., starvation), the body enters a catabolic state, resulting in enrichment of ^15^N and thus elevated δ^15^N. This pattern has been observed in modern populations using hair^41–43^ and in bioarchaeological populations using collagen from tooth dentine^44^. Studies using bone collagen to assess nutritional stress, however, have not observed elevated δ^15^N in individuals believed to have experienced chronic nutritional stress^44,45^. Analysis of rib bone collagen from medieval London revealed lower δ^15^N and no difference in δ^13^C in non-famine burials compared to contemporaneous famine burials^45^. The authors attributed this to the slow turnover rate in bone that averaged short-term changes in δ^15^N, arguing the results reflect differences in diet. Individuals most likely to suffer from the effects of a famine may be consuming a poorer quality diet not only during a famine, but throughout their lives. This is supported by Beaumont’s^46^ comparison of co-forming bone and dentine from archaeological juveniles that identified a potential ‘threshold’ for nutritional stress above which new bone is not formed; thus, short-term changes in δ^15^N and δ^13^C during a famine period would not be recorded in bone tissue.

Biochemical research regarding historical plague has largely focused on DNA analysis of *Y. pestis*. Unlike periods of famine, which are characterized by prolonged starvation, plague results in death within days, and thus does not produce isotopic signatures in bone and tooth tissue. For this reason, stable carbon and nitrogen isotope analyses are most useful for assessing differences in long-term diet among individuals who died or lived during outbreaks of plague, allowing nuanced interpretations of their social status based on their entitlement to certain foodstuffs, such as protein-rich food sources.

Though previous research provides insights on medieval crisis mortality patterns, there remain questions about the underlying mechanisms that might have produced the observed variation in risks of death, e.g., the extent to which variation was mediated by environmental conditions such as diet. Furthermore, previous bioarchaeological studies of medieval plague and famine did not account for missing data, which can substantially affect findings^47,48^. In this study, we build on previous research on plague and famine mortality patterns in medieval London by integrating stable isotopic data reflective of diet and by controlling for missing data.

### Aims and hypotheses

The goal of this study is to examine the differences in characteristics among people who died as a result of three categories of causes of death in medieval London: (1) famine, (2) plague of 1361, and (3) other causes. We examine demographic (i.e., sex, age-at-death), health/frailty (i.e., cribra orbitalia (CO), linear enamel hypoplasia (LEH), short femoral length), and dietary (i.e., δ^15^N and δ^13^C from bone collagen) indicators simultaneously to determine which factors are associated with elevated risk for the three categories of causes of death. Based on previous findings detailed above of (1) higher risks of mortality during the Black Death and plague of 1361 in London for those with skeletal stress markers, and (2) relatively high frequencies of developmental stress markers among victims of medieval famines, we hypothesize (Hypothesis 1) higher risks of mortality among individuals with skeletal stress markers in both medieval famine and plague of 1361 burials. We hypothesize (Hypothesis 2) higher risk of plague death with age among adults. Similarly, given evidence from recent famines that adults over the age of 45 years face high proportionate increases in mortality^49^we expect (Hypothesis 3) higher risk of famine death with age. We expect (Hypothesis 4) higher estimated risk of both famine and plague death for females. Finally, given analysis of δ^13^C and δ^15^N data from previous research by Walter and colleagues^45^ that includes cemeteries used in this study, we expect (Hypothesis 5) that individuals who experienced famine will exhibit lower δ^15^N and no difference in δ^13^C.

## Materials and methods

We evaluate data from 5405 adults (i.e., with estimated ages of death 18 years of age and older) from nine medieval London cemeteries recorded using the Wellcome Osteological Research Database (WORD) and curated by London Museum (Table 1): Bermondsey Abbey (BA84), Guildhall Yard (GYE92), Spital Square (NRT85, NRF88, SPQ88 and SSQ88), St. Mary Graces (MIN86), St. Nicholas Shambles (GPO75), Dominican Friary: Carter Lane (PIC87), Merton Priory (MPY86), St. Benet Sherehog (ONE94), and St. Mary Spital (SRP98). The cemeteries selected are meant to cover the scope of medieval life in London: all socio-economic groups (SES), all sexes, and both lay and religious individuals. More details about these cemeteries and the numbers of individuals from each included in this study are provided in the Supplementary Material. In addition to data from a collaborative project between authors SND and JB, we use stable isotope data from two other studies^50,51^. Osteological data were collected and the human remains were sampled for isotopic analysis with permission of, and in accordance with, the ethics and practice procedures described by London Museum Human Remains Policy^52^. We note the majority of individuals in our study are from St. Mary Spital; we discuss the possible influence of this on our findings below.

**Table 1:** Sample size from each cemetery included in this study.

Cemetery	Imputed *N*	δ^13^C and δ^15^*N* *N*
Bermondsey Abbey (BA84) (1066–1538 CE)	155	
Guildhall Yard (GYE92) (1050–1350 CE)	34	
Spital Square (NRT85, NRF88, SPQ88 and SSQ88) (1197–1280 CE)	29	
St. Mary Graces (MIN86) (1350–1538 CE)	194	68
St Nicholas Shambles (GPO85) (11–12th cen. CE)	195	70
Dominican Friary Carter Lane (PIC87) (1200–1538 CE)	28	
Merton Priory (MPY86) (1117–1538 CE)	468	
St. Benet Sherehog (ONE94) (1250–1500 CE)	12	
St Mary Spital (SRP98) (1120–1540 CE)	4290	244

### Sex, age, median date of cemetery samples, and frailty

Sex was estimated from features of the skull and pelvis and reported on a five-point scale of female, probable female, intermediate, probable male, or male; adults too fragmentary or poorly preserved for feasible sex estimation were scored as “undetermined”^53^. The individuals reported as “undetermined” or “intermediate” for sex in WORD were marked as missing data, and the “probable” categories were collapsed into the corresponding sex. We recognize sex is a continuum not reflected in conventional binary categories, but there is currently a lack of knowledge of how skeletal elements used to estimate sex might reflect intersex or nonbinary individuals. While it is not ideal to collapse sex into a binary variable, it allows for comparability across studies.

Transition analysis with Bayesian approach-derived age estimates were assigned to each individual in the dataset using the supplementary tables in Godde et al.^23^. Transition analysis removes bias that can be introduced by the known-age reference collections used to derive age standards, an issue called “age mimicry”^54^. There are two methods of transition analysis in the literature: (1) a component scoring method combined with transition analysis statistics e.g^54^.,, and (2) inputting the phases scored from traditional aging methods (e.g., Suchey-Brooks pubic symphysis method) into transition analysis statistics ^e.g., 55^. Godde et al.^23^ used the second approach and followed the protocol from Konigsberg et al.^55^ by inputting phase scores from the Suchey-Brooks pubic symphysis^56^ and Lovejoy et al.^57^ auricular surface aging methods into transition analysis statistics to produce ages-at-death specific to medieval London (see^23^ for information on the samples and statistics utilized to create their estimates). Here, we used the lookup tables created by Godde et al.^23^ to assign ages to the phases from Suchey-Brooks^56^ and Lovejoy et al.^57^ scored by the London Museum staff. The highest posterior densities (HPD), which reflect the most common age for an age indicator stage/phase, were assigned as point estimates for modeling in regression. As in Godde et al.^23^ and Godde and Hens^58^ the mean HPD rounded to the nearest year was used when phases from both aging methods were present. As approximately 300 years are represented with the data, median dates were calculated by cemetery from the range of dates assigned to the burials to control for differences over time.

Following the research design of DeWitte and Wood^22^ we use data on indicators of underlying frailty and health obtained from WORD and as recorded by museum staff, including CO, LEH, and short femur length as a proxy for stature. CO and LEH status were coded as present or absent from the WORD severity data. For individuals with isotope values who did not have CO scores in WORD, scores from the author SND were included. Short stature (extrapolated from short femur length) was coded as such if the measurement of maximum femur length was more than one standard deviation shorter than the sample mean.

### Missing data

Missing data (due to highly fragmented or missing skeletal elements) were estimated using the MissForest algorithm, which is a multiple imputation procedure for mixed (continuous and categorical) data that uses a Random Forest model^59^. The data for this study are missing not at random (MNAR), a situation for which MissForest outperforms other imputation methods in terms of accuracy^60^. Performance was evaluated via out-of-bag (OOB) error estimation using the normalized root mean squared error (NRMSE) for continuous variables and proportion of falsely classified entries (PFC) for categorical variables.

Missing data were predicted in four waves, taking into account contextual information and the pattern of missing data. Imputed variables included: sex, age-at-death, LEH presence/absence, CO presence/absence, femur length, and isotope ratios (δ^13^C and δ^15^N). The binary short femur and other frailty variables were created from measurements and severity scores after imputation.

For the first wave, as some information was known about status for all or some individuals in most cemeteries, we included only the cemeteries with this contextual information (all except St. Nicholas Shambles and St. Mary Spital) to create a complete dataset with a full cross-section of status for predicting missing data. Status was determined by whether an individual was buried in a monastic cemetery (wealthier individuals or those of probable religious status, e.g., monk or friar) or in a lower or higher status location within a cemetery (see details in the Supplementary Material). However, status was not included as a covariate in later statistical models due to a lack of information on status for burials at St. Nicholas Shambles and St. Mary Spital. Stable isotope ratios were not imputed in this step. In the second wave, the data excluding stable isotope ratios were imputed from the dataset constructed in wave one using information from lay cemeteries for St. Nicholas Shambles. For the third wave, the data for St. Mary Spital, excluding stable isotope ratios, were imputed from the full dataset constructed in waves one and two to capture information from across the other cemeteries, which was necessary due to the variety of statuses in this cemetery. Stable isotope ratios were imputed in the fourth and final wave using information from St. Nicholas Shambles, St. Mary Graces, and St. Mary Spital. As the statistical analyses in this paper focus on reporting characteristics of the group of individuals in the cemeteries and not information on particular individuals, imputation was deemed appropriate, as it will allow the creation of sufficiently large datasets that have similar properties to the original dataset with missing values for sophisticated statistical techniques.

### Stable isotope ratios (δ^13^C and δ^15^N)

Interpreting δ^13^C and δ^15^N of bone collagen from both faunal and human remains is a well-established method of reconstructing diet in past populations ^see 61^. Human bones contain collagen that has been laid down and remodeled over several years and thus represents an average of the protein in the diet that the body has used to synthesize that collagen over the growth period. The length of time represented varies depending on the skeletal element and the age of the individual^62^. Rib bone is thought to represent the last 5 years of life, while compact bone represents an average over a longer period, up to 10 years^63^. Advances in sampling strategies of dentine collagen from tooth samples^64^ have allowed the identification of variations in δ^13^C and δ^15^N during the growth of the tooth, revealing possible short-term changes that may be related to diet, migration, or even physiological stress^65–67^. In a British medieval population, based on previous studies, we would expect to see the consumption of a mixed diet consisting of terrestrial and marine resources e.g^68^. We are also aware that many religious orders had rules about diet^69^ and this will have influenced the isotope values seen in many of the individuals in this study. The relative δ^13^C and δ^15^N ratios in context with contemporaneous faunal data allow us to identify differences between individuals in their long-term diet^70^.

As mentioned previously, recent research has endeavored to go beyond diet reconstruction using stable isotope analysis to investigate potential physiological changes an individual may have experienced during life, such as undernutrition. The findings indicate that short-term variations in δ^13^C and δ^15^ N captured in dentine profiles from tooth samples appear to be related to physiological changes in an individual that result in the deposition of dentine collagen during a period of high nutritional demand (such as growth or chronic disease) or during a period of famine^44,65^. Though studies such as these have begun to uncover potential links between undernutrition and stable isotope signatures, it is unlikely that the bone collagen δ^13^C and δ^15^N data in our sample will exhibit these biochemical markers of nutritional stress given slow tissue turnover rates in rib bone tissue, the primary source of isotopic data for this study. Analysis of δ^13^C and δ^15^N for individuals interred in famine burials, however, may be informative of differences in diet that occurred as a consequence of their long-term nutrition during famine, rather than a physiological response due to starvation. Similarly, analysis of stable isotope data from individuals in plague burials would be informative of long-term diet for individuals who were at higher risk of death from plague.

For this study, we use δ^13^C and δ^15^N data primarily from rib bone samples, with the exception of samples from St. Mary Graces where all but three of the samples were from compact bone of long bones. All bones sampled showed no evidence of pathological bone formation and were prepared using a modified Longin method^71^. Only samples meeting quality requirements for collagen were used^[see 72–74^. The results are expressed using the delta (δ) notation in parts per thousand (per mil or ‰).

### Statistical analysis

To evaluate the demographic and health status differences between individuals who died of famine, the plague event of 1361 CE, and those who died from other causes, a common statistical approach in public health was applied to the data: multinomial logistic regression. The categorical outcome variable for the multinomial logistic regression is a measure of whether individuals died of famine, the plague of 1361 CE, or another cause of death. The multinomial logit calculates a relative risk ratio (RRR) for interpretation, which is a measure of association between the exposure (i.e., either age-at-death, sex, CO, LEH, short femur, δ^13^C, δ^15^N, or median date) and outcome (cause of death).

The type of multinomial regression used is a log-linear neural network-based algorithm, which outperforms the standard multinomial regression for reliability, accuracy, and finding statistical significance^75^. Akaike Information Criterion (AIC), log likelihood, and a McFadden’s pseudo R^2^ evaluated the fit of the multinomial logistic regressions and a variance inflation factor (VIF) verified there was no multicollinearity of variables. The logits were run using the nnet package (Venables and Ripley, 2002) in R^76^. Significance was judged at an alpha of less than 0.10, which should be sufficient for bioarchaeological studies for indicating a trend worthy of consideration. We selected this value a priori rather than a more conversative and conventional level because p-values are dependent on sample size, and when sample size is small (as is often a limiting factor in bioarchaeological studies), rejecting the null hypothesis (H_0_) when using a smaller alpha level can be nearly impossible if the size of the effect is not large.

As the continuous variables of age-at-death estimates and the stable isotope ratios violated the linearity to the log odds assumption of the logits, they were transformed to correct the issue. Age-at-death estimates and δ^15^N were transformed with log base 2, which allows for a simplified interpretation of the RRR (a doubling). Given that δ^13^C values are negative and therefore cannot be transformed, these were converted to a categorical variable representing quartiles (minimum = −22.1, 1 st quartile = −19.1, 2nd quartile = −19.0, 3rd quartile = −18. 9, maximum = −17.6). Two sets of multinomial models were run: (1) comparing the three types of causes of death to one another using all covariates, except δ^13^C and δ^15^N, which allowed for a comparison across all cemeteries (Model 1); and (2) comparing the three types of causes of death to one another using all covariates and also including an interaction term of sex and δ^13^C and δ^15^N to explore sex differences among stable isotopes ratios, which restricted the analysis to the three cemeteries with stable isotope data (Model 2).

## Results

The performance of MissForest for imputing missing data is excellent across all waves (Table 2), as the closer to zero an OOB estimate, the better the performance^59^. The descriptive statistics for each variable by cemetery post-imputation are included in Table 3. The VIFs for all models was less than 4. Some models had coefficients with large standard errors, indicating uncertainty in estimating that variable, and thus those coefficients are not interpreted. For readability, understanding, and alignment with our hypothesis, we further limit our interpretations of the RRR for continuous variables to an increase or a decrease in risk in relation to the reference group (e.g., as age-at-death increases there is also an increase in risk for individuals who died of plague as opposed to those who died of other causes), but we provide the RRR in the tables.

**Table 2:** Waves and OOB values. NRMSE measures continuous data and PFC, categorical data. st. Nicholas shambles is denoted with SNS and st. Mary spital with SMS.

	NRMSE	PFC
Wave 1: Includes lay cemeteries and status; excludes SNS, SMS, and isotopes	0.0231	0.0583
Wave 2: Includes SNS; excludes monks, SMS, isotopes, and status	0.0171	0.0411
Wave 3: Includes SMS; excludes isotopes	0.0239	0.0760
Wave 4: Includes isotopes	0.0007	NA

**Table 3:** Descriptive statistics (percentage or mean) of the imputed sample (*N* = 5,405).

Variable	famine (*n* = 2,005)	plague (*n* = 105)	another cause of death (*n* = 3,295)
Sex			
Female	51%	41%	38%
Male	49%	59%	62%
Age-at-Death	39	46	42
Cribra Orbitalia (CO)			
Present	46%	30%	39%
Absent	54%	70%	61%
Linear Enamel Hypoplasia (LEH)			
Present	51%	78%	50%
Absent	49%	22%	50%
Short Femur			
Present	12%	15%	11%
Absent	88%	85%	89%
Stable isotope ratios			
δ^13^C	−18.99	−19.4	−19.0
δ^15^N	12.5	12.1	12.7

The results of the model (1) that examined the covariates without δ^13^C and δ^15^N is found in Supplementary Material (Table S1). The McFadden’s pseudo R^2^ is poor^77^. In the first comparison, holding all other variables constant, individuals who died of famine (as opposed to other causes) were 1.5 times more likely to be female (vs. male), 1.3 times more likely to have CO (in comparison to not having CO), 14% less likely to have LEH (as opposed to no LEH), and were younger (vs. someone else who died of other causes). Short femur had no significant relationship with cause of death. For the second analysis, when holding the covariates constant, individuals who died of plague (as compared to other causes) were 1.3 times more likely to be female (as opposed to male), 25% less likely to have CO (rather than none), 3.9 times more likely to have at least one LEH (vs. no LEH), 1.3 times more likely to have a short femur (in comparison to average length), and were older (as opposed to someone else who died of other causes). In the third comparison of dying of the plague vs. dying of famine, individuals dying of plague were 15% less likely to be female, individuals were 42% less likely to have CO, 4.5 times more likely to have at least one LEH (as compared to none), were 1.3 times more likely to have a short femur (as opposed to average length), and were older (as compared to individuals dying of famine). Median date was significant in all three of these comparisons, indicating there were differences across time.

Model 2 (Table 4) examines how the addition of δ^13^C and δ^15^N affected the profiles of those who died of famine, plague, and other causes. The AIC is lower than that for Model 1 and the log likelihood is higher, while the McFadden’s pseudo R^2^ is excellent, which together indicates the addition of δ^15^N and δ^13^C data better discriminated among the causes of death. In the first comparison, holding all other covariates constant, individuals who died of famine (as opposed to other causes) were less likely to be female (vs. male; RRR is near zero), 1.1 times more likely to have CO (vs. not having CO), 24% less likely to have a short femur (vs. average length), and were younger (in relation to someone else who died of other causes). With respect to δ^13^C and δ^15^N, when holding the other variables constant, individuals who died of famine were 1.2 (fourth quartile), 3.3 (third quartile), and 1.8 (second quartile) times more likely to have higher δ^13^C (vs. first quartile), and more likely to have lower δ^15^N. The interactions of sex with δ^13^C and δ^15^N revealed, when the other variables are accounted for, females who died of famine were more likely to have third quartile (51–75% of the data) and second quartile (26–50% of the data) δ^13^C as opposed to first quartile, and higher δ^15^N. Other variables, except median date, were not significant.

When the covariates from the second comparison in Model 2 were held constant, individuals who died of plague (as opposed to those who died of another cause) were less likely to be female (in comparison to males; RRR is near zero), 6.6 times more likely to have at least one LEH (as opposed to none), be older (in comparison to someone else who died of another cause), less likely to have δ^13^C in the second and third quartiles as opposed to the first quartile, higher fourth quartile δ^13^C values (in comparison to the first quartile) and lower δ^15^N. The interaction terms of sex with δ^15^N and δ^13^C show that when other variables are controlled, females who died of the plague had lower fourth quartile δ^13^C (vs. first quartile) and lower δ^15^N. No other variables were significant, except median date.

The contrast of individuals who died of plague to those who died of famine (third comparison within Model 2) generally supports the prior two comparisons’ results: plague victims were 5.9 times more likely to have at least one LEH (as opposed to none), were older, and have lower δ^13^C and δ^15^N. The interaction terms yielded results that indicate when the covariates are held constant, females who died of plague were less likely to have fourth quartile δ^13^C values in comparison to first quartile. Median date was the only other significant variable and was significant across the comparisons, indicating there were changes across time for which we controlled.

**Table 4:** Model 2: multinomial logistic regression models with isotopes.1 statistically significant results are shown in bold.

Models	Covariate	B	SE	*p*	RRR
Famine vs. another cause of death	Intercept	69.9082	11.1281	**< 0.0001**	2.29E + 30
	Sex (female)	−29.3239	−3.9394	**< 0.0001**	1.84E-13
	CO (present)	0.1227	1.8453	**0.0650**	1.1305
	LEH (present)	0.0991	1.4783	0.1393	1.1042
	Short Femur (present)	−0.2780	−2.7259	**0.0064**	0.7573
	Age-at-Death (log base 2)	−0.2413	−4.3261	**< 0.0001**	0.7856
	δ^13^C (Fourth Quartile)	2.4997	13.5824	**< 0.0001**	1.1973
	δ^13^C (Third Quartile)	0.8973	6.1286	**< 0.0001**	3.2662
	δ^13^C (Second Quartile)	0.5917	4.5731	**< 0.0001**	1.7784
	δ^15^N (log base 2)	−24.3158	−15.4550	**< 0.0001**	2.75E-11
	Sex (female): δ^13^C (Fourth Quartile)	0.1800	0.7124	0.4762	1.1973
	Sex (female): δ^13^C (Third Quartile)	1.1836	5.4737	**< 0.0001**	3.2662
	Sex (female): δ^13^C (Second Quartile)	0.5757	2.6723	**0.0075**	1.7784
	Sex (female): δ^15^N (log base 2)	7.8656	3.8185	**0.0001**	2606.0370
	Median Date (log base 2)	1.8216	5.1269	**< 0.0001**	6.1818
Plague vs. another cause of death	Intercept	−10.9652	−0.6898	0.4904	1.73E-05
	Sex (female)	−32.3237	−2.5180	**0.0118**	9.16E-15
	CO (present)	0.0078	0.0311	0.9752	1.0078
	LEH (present)	1.8797	7.2188	**< 0.0001**	6.5518
	Short Femur (present)	0.1554	0.4991	0.6177	1.1681
	Age-at-Death (log base 2)	1.3580	6.6716	**< 0.0001**	3.8885
	δ^13^C (Fourth Quartile)	0.9309	1.8060	**0.0709**	2.5367
	δ^13^C (Third Quartile)	−1.4480	−2.5255	**0.0116**	0.2350
	δ^13^C (Second Quartile)	−1.4958	−2.9071	**0.0036**	0.2241
	δ^15^N (log base 2)	−31.9541	−11.1386	**< 0.0001**	1.33E-14
	Sex (female): δ^13^C (Fourth Quartile)	−1.4578	−1.8583	**0.0631**	0.2328
	Sex (female): δ^13^C (Third Quartile)	0.4530	0.5548	0.5790	1.5730
	Sex (female): δ^13^C (Second Quartile)	0.5952	0.7674	0.4428	1.8134
	Sex (female): δ^15^N (log base 2)	−31.9541	2.4828	**0.0130**	7065.8370
	Median Date (log base 2)	11.1736	8.0908	**< 0.0001**	71228.4142
Plague vs. famine cause of death	Intercept	−80.8845	−5.2456	**< 0.0001**	7.45E-36
	Sex (female)	−2.9869	−0.2611	0.7940	0.0504
	CO (present)	−0.1149	−0.4591	0.6462	0.8915
	LEH (present)	1.7807	6.8288	**< 0.0001**	5.9339
	Short Femur (present)	0.4333	1.3943	0.1632	1.5424
	Age-at-Death (log base 2)	1.5993	7.8542	**< 0.0001**	4.9497
	δ^13^C (Fourth Quartile)	−1.5690	−3.0947	**0.0020**	0.2083
	δ^13^C (Third Quartile)	−2.3452	−4.0751	**< 0.0001**	0.0958
	δ^13^C (Second Quartile)	−2.0875	−4.0406	**< 0.0001**	0.1240
	δ^15^N (log base 2)	−7.6358	−2.9784	**0.0029**	0.0005
	Sex (female): δ^13^C (Fourth Quartile)	−1.5690	−2.0851	**0.0371**	0.1945
	Sex (female): δ^13^C (Third Quartile)	−2.3452	−0.8877	0.3747	0.4817
	Sex (female): δ^13^C (Second Quartile)	−2.0875	0.0252	0.9799	1.0200
	Sex (female): δ^15^N (log base 2)	−7.6358	0.3111	0.7557	2.7016
	Median Date (log base 2)	9.3522	6.7914	**< 0.0001**	11524.6200

## Discussion

The profiles of individuals who died of famine, plague of 1361, and other causes of death are very different. The models without stable isotope data (Supplementary Material Table S1), which represent individuals across all cemeteries included in this study, suggest (1) individuals who died of famine were more likely to be female, be younger than those who died of other causes, have CO lesions, and were less likely to have LEH; and (2) individuals who died of plague were more likely to be female (as opposed to other causes, but less likely than those who died of famine), be older, less likely to have CO lesions, more likely to have at least one LEH, and have a short femur. When stable isotope results were added to the models (Table 4), the profiles changed slightly: (1) individuals who died of famine were more likely to be male, younger, have CO lesions, have an average or longer femur than other causes, higher δ^13^C, and lower δ^15^N; and (2) individuals who died of plague were more likely to be male, be older, have at least one LEH, a mix of lower and higher δ^13^C, and lower δ^15^N. The models did not perform as well without isotope data, indicating sex, age-at-death, and frailty markers were not strong differentiators among causes of death; in other words, there was a large amount of heterogeneity that was not explained by these variables. However, not surprisingly, the isotope variables more easily discriminated among causes of death. By examining patterns in light of stable isotope data informative about diet, we may reveal otherwise hidden heterogeneity in frailty associated with dietary variation and, more generally, access to resources shaped by social position and identity in this context.

We focus the remainder of our discussion on the result of the model that included dietary stable isotopes (Model 2, Table 4), as it performed better. The results generally support our hypothesis of higher risks of famine and plague mortality for individuals with skeletal stress markers (Hypothesis 1). In particular, based on examination via multinomial logistic regression, CO and a short femur appear to be significantly linked to famine death, and LEH to plague death. Similarly, the results are consistent with our prediction that adults who died of plague are more likely to be older than younger (Hypothesis 2). However, the results do not support our prediction that famine victims are more likely to be older adults compared to younger adults (Hypothesis 3). The results suggest that both famine and plague victims were more likely to be male and thus do not support our hypotheses of higher risks of famine death for females (Hypothesis 4) based on previous bioarchaeological research in the context of medieval London.

### Famine

The stable isotope analysis results provide partial support for Hypothesis 5. The results are consistent with previous research that revealed lower δ^15^ ^15^N in famine burials compared to non-famine burials in St. Mary Spital cemetery^45^ and thus support Hypothesis 5 with respect to nitrogen. However, contrary to previous research^45^ we observe a difference in δ^13^C, with higher δ^13^C in famine burials. This is likely due to the larger sample size included in our study, allowing the slight difference in δ^13^C to be captured. The lower δ^15^N and higher δ^13^C for famine burials suggest a lower trophic-level diet with some marine input over a prolonged period. Thus, individuals who died from famine consumed less high trophic-level protein, such as animal meat and marine fish, compared to those who died from other causes.

Rawcliffe^78^ notes that individuals in London during non-famine periods would have had better access to fish and terrestrial animal proteins (high trophic-level foods) compared to individuals experiencing famine. However, there is some evidence of inequality of access to fish in diets as evidenced in historical documentation^79^: p^17^. and stable isotope analyses^79^ in medieval York, with women eating fewer marine resources. Given that the medieval English diet is largely C_3_-based with some animal and fish protein^68,80^ it is likely that during periods of famine, individuals could not supplement their diet with animal meat and/or fish (marine protein) due its limited or fluctuating availability in the market. Limited access to natural water sources meant Londoners had few opportunities to supplement their diets through fishing. Although the Thames was used for fishing during this period, it was primarily used for commercial operations^81^. As urban residents, they also had little access to hunting wild game, relying instead on markets and street vendors for most of their protein^82^. During this period there were also documented decades-long instances of cattle plagues that caused beef and dairy shortages^35^ which would have affected access to high protein foods, particularly for the poor who relied on dairy products as a main source protein^83^. Müldner et al.^84^ observed a similar pattern in δ^15^N and δ^13^C between high- and low-status individuals in medieval Scotland, with low-status individuals exhibiting a similar diet to famine individuals in this study. Historical records support this, noting that, in medieval England, high-status individuals consumed more animal meat and fish compared to the poor^85^.

The lower proportion of protein consumed by individuals facing famine in this study could have increased their susceptibility to infection and eventually death. Studies in both human and animal models have concluded that protein malnutrition influences the immune system^86,88,88^. Curto et al.^89^ found that archaeological individuals with non-specific generalized infections had lower δ^15^N compared to those without lesions and those only with healed tibial periostitis. They argue that those individuals with skeletal indicators of infection at death consumed less animal protein, increasing their susceptibility to pathogens that led more frequently to generalized infections.

Our finding that famine victims were more likely to be male is not consistent with previous bioarchaeological findings by DeWitte and Yaussy^40^ of lower hazards of death for males under famine conditions following the Black Death and does not support our Hypothesis 4. However, it is consistent with observations that males frequently experienced the highest risks of death during famine events over the last 250 years^49,90,92–94,94^. Such findings may reflect enhanced physiological buffering of females because of their typically higher fat stores (energy stores to mobilize during dietary deficiencies) and lower muscle mass (which is a metabolically expensive tissue)^91,93^. Further, female advantages during famine conditions may reflect the generally immune-enhancing effects of estrogen, an important factor given that deaths during famine events are most often caused by infectious disease rather than starvation per se^95^.

Our finding that LEH do not appear to be significantly linked to famine death is not consistent with previous findings by Yaussy et al.^39^; however, our analyses also reveal a significant effect of CO on famine death that was not discerned by previous work. In general, our findings indicate that developmental stress is associated with greater vulnerability to famine death later in life. This association may reflect the long-term negative effects of modifications of developmental trajectories that provide a short-term survival advantage during childhood, as predicted by the Developmental Origins of Health and Disease hypothesis^96,97^. For example, nutritional stress during childhood may induce an energetic trade-off that impairs the immune system, leading to greater vulnerability to infectious disease (the leading cause of death during famines) across the lifespan^98,100–102,102^. Alternatively, individuals with evidence of developmental stress might have lived in persistent or repeated conditions of deprivation, such that the association between developmental stress markers and famine mortality reflects poor living conditions rather than a mechanistic effect of early life stress on mortality in adulthood.

### Plague

The enhanced performance of the model with the inclusion of stable isotope ratios indicates that there is likely some underlying dietary factor associated with plague mortality. Individuals with elevated mortality exhibit lower δ^15^N and a mixture of higher and lower δ^13^C. The lower δ^15^ ^15^N in plague victims overall could be related to a lower proportion of animal protein in the diet, whether through dietary choice or food insecurity, which may have increased disease susceptibility as discussed for the famine analyses above. When comparing the sexes, males, who were more likely to die from plague compared to females, exhibit lower δ^15^N. This may be due to males having poorer access to animal protein sources during plague, though differential access to animal meat in favor of females in medieval England is not, to our knowledge, documented in the literature. It is thus more likely that males and females consumed similar diets, but females were better buffered from the detrimental effects of a low protein diet due to their tendency to maintain higher fat stores and lower muscle mass as discussed above.

Our finding that individuals who died of plague were more likely to be male does not support our Hypothesis 4, but is consistent with reports of some chroniclers of the plague of 1361 CE suggesting men were disproportionately affected by the epidemic^103,104^. Further, our observation that plague victims were older than individuals who died of other causes supports our Hypothesis 2 and is consistent with previous bioarchaeological findings for the Black Death ^but see 23,26^. We find an association between the presence of a developmental stress marker (LEH) and plague death, as was found previously for the Black Death^22,23^. We suggest that these associations may reflect the same underlying mechanisms and factors detailed for famine above.

As noted above, our sample is biased toward individuals from the St. Mary Spital cemetery. Thus, we cannot discount the possibility that the predominance of individuals from St. Mary Spital cemetery in our sample means that our model estimates are strongly driven by patterns at that site. However, as people with a variety of identities were interred there, its influence on our estimates aligns with our broader study design, which mitigates concerns about uneven sampling across the cemeteries. We also note that across all causes of death (plague, famine, and other causes), our samples have biased sex ratios in favor of males. While we do not have any independent information (e.g., from contemporary historical records) to determine whether there was a sex/gender-bias in who was provided burial in the catastrophic (plague and famine) burials nor in the lay burials, many of the “other causes” burials included in our study are from monastic cemeteries that were predominated by male religious personnel, and that may drive at least some of the skewed sex ratio in our samples. The second author previously observed male-biased sex ratios for medieval Danish urban cemeteries and a London Black Death (*c.* 1349) cemetery (East Smithfield) and suggested this might reflect real imbalances in sex ratios in the once living populations (perhaps driven by factors such as disproportionate migration by men seeking employment in urban centers). Importantly, previous work^105^ found that biased sex ratios did not affect estimates of sex differences in hazards of death.

In general, the findings of our study highlight how various biological and social factors shaped risks of mortality in medieval London, even in the context of major mortality crises, often assumed to be indiscriminate in nature. While we have controlled for as many documentable variables affecting the outcome variables as possible with the available data, there are certainly others for which we cannot account. As is typical in bioarchaeological studies, there are many potential hidden sources of heterogeneity among the individuals included in our study, including, but not limited to migration, occupation, and exposures to environmental toxicants. Future work integrating additional data on migrant status and high-resolution data on exposure to severe nutritional stress *in utero* and in childhood may help further clarify other variables that interacted with those considered in this study to either exacerbate or reduce risks of death in the past during crisis mortality events.

## Supplementary Information

Below is the link to the electronic supplementary material.

Supplementary Material 1

Supplementary Material 2

## Data Availability

All data used in this study are available in the supplementary materials.
