# Supplementary material for: Selective mortality during famine and plague events in medieval London
Source: Sci Rep. 2025 Jul 25;15:27133. doi: 10.1038/s41598-025-13198-7 (PMC12297707; doi:10.1038/s41598-025-13198-7)
Supplement: Supplementary file 2 — Supplementary Material 2 [file 41598_2025_13198_MOESM2_ESM.docx]

**Supplementary Material: ­­**

**Selective mortality during famine and plague events in medieval London**

Godde et al.

**Cemeteries included in this study:**

The Cluniac Priory of St. Saviour Bermondsey, Southwark (Bermondsey Abbey, c. 1066–1538 CE) was established in the 1080s CE south of the River Thames. The Augustinian Priory of St. Mary Merton, Surrey (Merton Priory, c. 1117­–1538 CE) was established in c. 1114–1117 CE in a rural area in the valley of the River Wandle ^1^. The Merton Priory and Bermondsey Abbey cemeteries, though primarily monastic, may contain lay individuals. Many medieval monasteries allowed burial of lay people in their cemeteries in exchange for bequests of land or money, which would generally exclude poor people from the monastic samples ^2,3^. The Carter Lane cemetery derive from the ruins of a Norman fortress (1050–1200 CE) that the Dominicans bought in 1274 or 1276 CE for the stone to build their nearby friary. Once this work was completed, the area was used as a burial ground between the 13^th^ and 14^th^ centuries CE ^4^.

The Guildhall Yard site (c. 1050­–1350 CE) was the site of the lay cemetery for St. Lawrence Jewry ^4^. St. Benet Sherehog (c. 1250–1500 CE) was a small but comparatively affluent parish. It was regularly a contributor to poor relief, and average rents within the parish were quite high compared to the city average ^5^.

The Cistercian Abbey of St. Mary Graces (c. 1359–1538 CE) was established just after the Black Death ended in London in 1350 ^6,7^. It is possible to determine the status of individuals buried at St. Mary Graces based on burial location. Lay individuals of lower status were interred in a cemetery associated with the Abbey, and monks and higher status lay people were buried within the Abbey’s church and chapels ^6,8,9^. There were victims of 14^th^-century plague (possibly the *pestis secunda* of 1361 CE in an area spatially distinct from the rest of the St. Mary Graces burials ^2,10^.

The hospital and priory of St. Mary Spital was founded by a group of wealthy London merchants in 1197 CE in response to the City’s rapidly increasing population and growing need to provide charity for the deserving poor, pilgrims, and pregnant women ^11^. However, there is a greater representation of the London population than might be inferred from these founding motivations – i.e., it is not a paupers’ cemetery. In addition to the people who lived and were cared for by the institution, the cemetery was used for the general population of London and higher-status benefactors of the institution, and it is considered to primarily be a secular cemetery ^11^. The cemetery has both single and small multiple burials, and mass burials, which correspond to famine events ^11^.

St. Nicholas Shambles was a small parish church established in 1144 or 1187 CE and closed in 1548–51 CE. Burials date to the 11^th^ and 12^th^ centuries CE ^12–14^. All but two of the individuals were buried in single graves; the only double grave was of a young adult female who had died in childbirth (sks 5061 and 5062).

Just as the monastic cemetery samples may include lay individuals, the lay cemeteries in this study (e.g., St. Mary Graces), also contain monks and other individuals of religious status, though the majority of burials represent non-monastic individuals ^8^.

**References**

1. Miller, P. & Saxby, D. *The Augustinian Priory of St Mary Merton, Surrey. Excavations 1976-1990.* (Museum of London, London, 2007).

2. Gilchrist, R. & Sloane, B. *Requiem: The Medieval Monastic Cemetery in Britain*. (Museum of London Archaeology Service, London, 2005).

3. Mays, S. The osteology of monasticism in medieval England. in *Social Archaeology of Funerary Remains* (eds. Gowland, R. & Knüsel, C.) 179–189 (Oxbow Books, Oxford, UK, 2006).

4. Schofield, J. & Maloney, C. *Archaeological Excavations in the City of London 1907-91*. (Museum of London, 2021).

5. Miles, A., White, W. & Tankard, D. *Burial at the Site of the Parish Church of St Benet Sherehog before and after the Great Fire: Excavations at 1 Poultry, City of London.* (Museum of London Archaeology Service, London, 2008).

6. Grainger, I. & Hawkins, D. Excavations at the Royal Mint site 1986-1988. *Lond. Archaeol.* **5**, 429–436 (1988).

7. Grainger, I., Hawkins, D., Cowal, L. & Mikulski, R. *The Black Death Cemetery, East Smithfield, London. Museum of London Archaeology Service Monograph 43.* (Museum of London Archaeology Service, London, 2008).

8. Grainger, I. & Phillpotts, C. *The Cistercian Abbey of St Mary Graces, East Smithfield, London. MoLA Monograph 44.* (Museum of London Archaeology, London, 2011).

9. Rogers, J. & Waldron, T. DISH and the monastic way of life. *Int J Osteoarch* **11**, 357–365 (2001).

10. Bos *et al.* Eighteenth century Yersinia pestis genomes reveal the long-term persistence of an historical plague focus. *eLife* **5**, e12994 (2016).

11. Connell, B., Jones, A., Redfern, R. & Walker, D. *A Bioarchaeological Study of Medieval Burials on the Site of St. Mary Spital*. (Museum of London Archaeology, London, 2012).

12. Dyson, T. *The Cemetery of St Nicholas Shambles*. (London and Middlesex Archaeological Society, London, 1988).

13. Schofield, J. *The Cemetery of St Nicholas Shambles*. (London and Middlesex Archaeological Society, London, 1988).

14. Rivière, S. Archaeological evidence for the church of St Nicholas, survival of strata and date of the cemetery, and burial types and burial practices. in *Skeletal remains from the cemetery of St Nicholas Shambles, City of London* (eds. In, W. & W.) 8–27 (London and Middlesex Archaeological Society, London, 1988).

| **Table S1.** Model 1: multinomial logistic regression model without isotopes.^1^ Statistically significant results are shown in bold. | | | | | | |
| --- | --- | --- | --- | --- | --- | --- |
|  |  |  |  |  |  | |
| **Models** | **Covariate** | **B** | **SE** | **p** | **RRR** | |
| **Famine vs. Another Cause of Death** |  |  |  |  |  | |
|  | Intercept | 1.9539 | 0.0018 | **<0.0001** | 7.0559 | |
|  | Sex (female) | 0.4363 | 0.0570 | **<0.0001** | 1.5469 | |
|  | Age-at-Death (log base 2) | -0.0118 | 0.0018 | **<0.0001** | 0.9883 | |
|  | CO (present) | 0.2561 | 0.0584 | **<0.0001** | 1.2919 | |
|  | LEH (present) | -0.1528 | 0.0581 | **0.0085** | 0.8583 | |
|  | Short Femur (present) | -0.0158 | 0.0899 | 0.8607 | 0.9843 | |
|  | Median Date (log base 2) | -0.0017 | 0.0001 | **<0.0001** | 0.9983 | |
| **Plague vs. Another Cause of Death** |  |  |  |  |  | |
|  | Intercept | -14.6819 | 0.0001 | **<0.0001** | 4.20E-07 | |
|  | Sex (female) | 0.2636 | 0.0017 | **<0.0001** | 1.3016 | |
|  | Age-at-Death (log base 2) | 0.0180 | 0.0061 | **0.0030** | 1.0181 | |
|  | CO (present) | -0.2912 | 0.0015 | **<0.0001** | 0.7473 | |
|  | LEH (present) | 1.3494 | 0.0012 | **<0.0001** | 3.8550 | |
|  | Short Femur (present) | 0.2595 | 0.0033 | **<0.0001** | 1.2963 | |
|  | Median Date (log base 2) | 0.0072 | 0.0002 | **<0.0001** | 1.0072 | |
| **Plague vs. Famine Cause of Death** |  |  |  |  |  | |
|  | Intercept | -16.6348 | 6.45E-05 | **<0.0001** | | 5.96E-08 |
|  | Sex (female) | -0.1726 | 0.0031 | **<0.0001** | | 0.8415 |
|  | Age-at-Death (log base 2) | 0.0298 | 0.0062 | **<0.0001** | | 1.0302 |
|  | CO (present) | -0.5474 | 0.0028 | **<0.0001** | | 0.5785 |
|  | LEH (present) | 1.5019 | 0.0024 | **<0.0001** | | 4.4903 |
|  | Short Femur (present) | 0.2753 | 0.0065 | **<0.0001** | | 1.3170 |
|  | Median Date (log base 2) | 0.0088 | 0.0002 | **<0.0001** | | 1.0089 |
| ^1^AIC: 7811.39, McFadden’s Pseudo R^2^: 0.035, Log Likelihood: -3891.69 | | | | | | |
